# Immune characteristics of severe and critical COVID-19 patients

**DOI:** 10.1038/s41392-020-00296-3

**Published:** 2020-08-31

**Authors:** Li Yang, Jianjun Gou, Jianbo Gao, Lan Huang, Zhiqiang Zhu, Shaofei Ji, Hongchun Liu, Lihua Xing, Mengying Yao, Yi Zhang

**Affiliations:** 1 Biotherapy Center, The First Affiliated Hospital of Zhengzhou University, 450052 Zhengzhou, Henan China; 2 Department of Clinical Laboratory, The First Affiliated Hospital of Zhengzhou University, 450052 Zhengzhou, Henan China; 3 Department of Radiology, The First Affiliated Hospital of Zhengzhou University, 450052 Zhengzhou, Henan China; 4 Department of Emergency, The First Affiliated Hospital of Zhengzhou University, 450052 Zhengzhou, Henan China; 5 Department of Radiology, Orthopaedic Hospital of Zhengzhou City, 450052 Zhengzhou, Henan China; 6 Department of Respiratory, The First Affiliated Hospital of Zhengzhou University, 450052 Zhengzhou, Henan China

**Keywords:** Medical research, Diseases

**Dear Editor**,

Recently, the novel coronavirus disease (COVID-19) has broken out worldwide,^1^ with rapid increase of infected patients. COVID-19 dominantly leads to pneumonia.^2^ Among these COVID-19 patients, some appears to be severe symptoms with acute respiratory distress syndrome, organ failure,^2^ and further present a poor outcome.

Previous studies have been reported that immune patterns are closely associated with disease progression of patients infected with other viruses.^3^ The correlation between immune signatures and outcome of severe and critical COVID-19 cases was not well illuminated. Therefore, we aimed to evaluate the correlation between immune characteristics, especially levels of lymphocytes and cytokines in peripheral blood, and clinical parameters in severe and critical COVID-19 patients, in order to find critical indicators of disease progression and to provide important guides for therapeutic strategy.

Thirty-six adult cases with severe and critical COVID-19 were enrolled. The disease outcome, immune patterns, microbiota infection, coagulation profile, and organ dysfunction biomarkers were analyzed and collected. This project was approved by the Ethics Committee of our hospital (No. 2020-KY-060), and all patients signed the informed consent.

We found that the cell numbers of lymphocytes in these patients were obviously decreased compared to that in healthy donors, including total lymphocytes, total T, CD4^+^ T, CD8^+^ T, B and NK cells (Fig. 1a), and the percentage of lymphocytes in COVID-19 patients was also significantly decreased except B cells (Supplementary Fig. S1a, b), with an increased ratio of CD4^+^/CD8^+^ T cells (Supplementary Fig. S1c), suggesting that CD8^+^ T cell number was more decreased than CD4^+^ T cell number in these patients. Particularly and importantly, the mean value of only B cell number (150.05/μL) was within normal range [(90–560)/μL] (Fig. 1a). All these data indicate that lymphocytes are impaired in severe and critical COVID-19 patients, especially more impaired in CD8^+^ T cells than in CD4^+^ T cells, and little impaired in B cells, which results in the higher percentage of B cells.

**Fig. 1:**
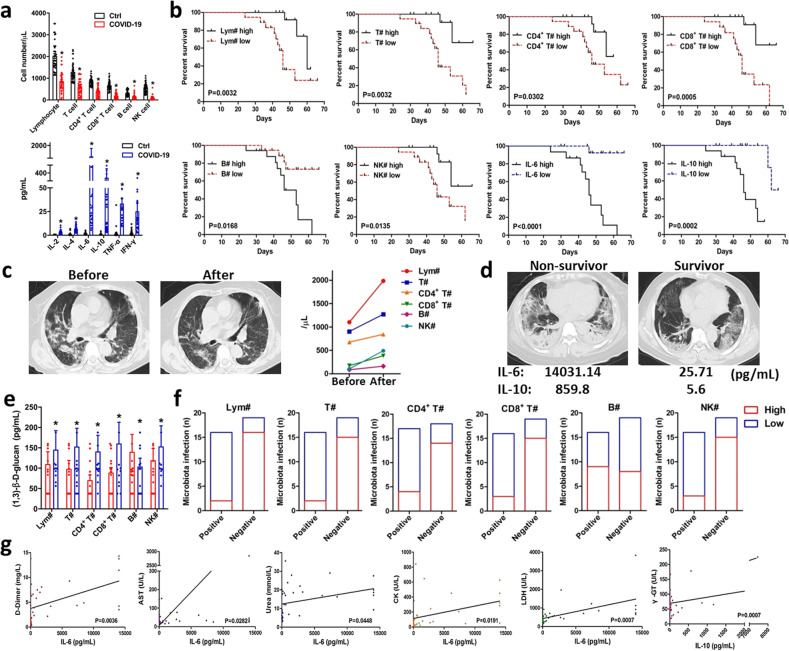
Immune characteristics are closely correlated with disease progression of COVID-19. **a** Lymphocyte subpopulations in the peripheral blood of COVID-19 (*n* = 36) and control (healthy donor, *n* = 36) groups were evaluated by flow cytometry. Cell numbers of total lymphocytes, total T, CD4^+^ T, CD8^+^ T, B, and NK cells were analyzed. Levels of cytokines (IL-2, IL-4, IL-6, IL-10, TNF-α, and IFN-γ) in the peripheral blood of COVID-19 and control (healthy donor, *n* = 36) groups were evaluated by flow cytometry, presented as a histogram. **b** Kaplan–Meier survival curves for 36 severe and critical COVID-19 patients with high and low levels of lymphocyte subpopulations, IL-6 and IL-10. **c** CT imaging matched with lymphocyte subpopulations from one representative patient during disease recovery. Before: disease severe stage; After: disease recovery stage. **d** CT imaging matched with IL-6 and IL-10 levels from one non-survivor and one survivor. **e** (1,3)-β-D-glucan in patients with high and low levels of lymphocyte subpopulations was analyzed by G test. **f** Patient numbers with high and low lymphocyte subpopulations infected with microbiota. **g** The relationships between IL-6 and D-Dimer, AST, Urea, CK, and LDH, or IL-10 and γ-GT were analyzed. Data are represented as means ± standard error (SE). ns nonsignificant, *P < 0.05

Furthermore, the cytokine levels, including IL-2, IL-4, IL-6, IL-10, TNF-α, and IFN-γ, were remarkably increased; especially IL-6 and IL-10 (Fig. 1a, Supplementary Fig. S2a). The results presented that the maximum values of IL-2, IL-4, IL-6, IL-10, TNF-α, and IFN-γ were 16.66, 16.87, >14031.14, 7451.48, 155.53, and 453.95 (pg/mL), respectively (Supplementary Fig. S2b). Moreover, the percentages of patients with IL-6 and IL-10 upregulation were 97.0% and 100.0%, respectively, which were significantly higher than that of patients with other cytokine upregulation (Supplementary Fig. S2b). In addition, the levels of IL-6 and IL-10 in critical COVID-19 patients were significantly higher than that in severe COVID-19 patients (Supplementary Fig. S2c). These findings demonstrate that cytokine level was elevated in severe and critical COVID-19 patients, particularly IL-6 and IL-10 were enormously increased.

To further evaluate the correlation between these immune parameters and clinical prognosis, we analyzed the overall survivals in patients with high and low levels of lymphocyte subpopulations and cytokines. We found that patients with high levels of total lymphocytes, total T, CD4^+^ T, CD8^+^ T, and NK cells had a good survival [cutoff value: total lymphocytes, 824.08; total T, 593; CD4^+^ T, 376; CD8^+^ T, 191; NK, 82.24 (/μL)] (Fig. 1b). Furthermore, patients with high levels of IL-6 and IL-10 had a poor overall survival [cutoff value: IL-6, 65.918; IL-10, 8.43 (pg/mL)] (Fig. 1b). Among these COVID-19 patients, the percentages of patients with high levels of lymphocytes, including total lymphocytes, total T, CD4^+^ T, CD8^+^ T, and NK cells, were obviously higher in all survivors than that in the non-survivors (Supplementary Fig. S3a), and there was an opposite result for B cells. Meanwhile, patients with low levels of IL-6 and IL-10 were basically live (Supplementary Fig. S3b). Through computed tomography (CT) imaging of one patient during the recovery, inflammation was markedly reduced, which accompanied by the increased levels of each lymphocyte subpopulations (Fig. 1c). Furthermore, one non-survivor presented a severe inflammation by CT imaging with serious IL-6 and IL-10 production; however, in another survivor, IL-6 and IL-10 levels were significantly decreased with a relatively mild inflammation by CT imaging (Fig. 1d). Therefore, immune characteristics are closely associated with disease progression, which could be served as a potential biomarker for prognosis of severe and critical COVID-19 patients.

It has been shown that there is a close crosstalk between immune homeostasis and gut microbiota on host in several diseases. So we further investigated the effect of lymphocytes on infection of microbiota in these COVID-19 patients, which could accelerate disease progression. (1,3)-β-D-glucan, a key structural component of fungus cell wall, in patients with low levels of lymphocytes, was significantly higher than that in patients with high level lymphocytes, excluded B cells (Fig. 1e). Most of the patients infected with microbiota had a lower level of lymphocytes, also excluded B cells (Fig. 1f). The special gut microbiota, which were infected in severe and critical COVID-19 patients, included baumanii, klebsiella pneumonia, enterobacter cloacae complex, stenotrophomonas maltophilia, candida famata, candida glabrata, candida albicans, enterococcus, burkholderia cepacia, pseudomonas aeruginosa, and saccharomyces cerevisiae (Supplementary Table S1). And baumanii was the most frequent microbiota that infected patients (Supplementary Table S1). Collectively, low lymphocyte level in COVID-19 patients is closely associated with microbiota infection.

Lastly, IL-6 level was closely correlated with D-Dimer and organ dysfunction biomarkers [liver: aspartate aminotransferase (AST), kidney: urea, heart: creatine kinase (CK) and lactate dehydrogenase (LDH)] (Fig. 1g). With IL-10 increase, γ-glutamyl trans peptidase (γ-GT) level (liver dysfunction) was obviously upregulated (Fig. 1g). The data demonstrate that elevated cytokine level is closely associated with severe syndromes of blood coagulation and multiple organs dysfunction.

In this study, severe and critical COVID-19 patients exhibit lymphopenia and high level of cytokines, especially impaired T cells, and increased IL-6 or IL-10, which are served as potential biomarkers for disease progression. Other studies also reported that lymphocyte deficiency or incapacity in COVID-19 patients promoted disease progression,^4,5^ and most of severe cases presented elevated levels of infection-related biomarkers and inflammatory cytokines.^5^ Low level of lymphocytes was closely related with microbiota infection in these patients, suggesting that impaired lymphocytes further induced microbiota infection to exacerbate disease progression, which could provide a guide for the antibiotic usage of severe and critical patients with microbiota infection. The production of a large number of inflammatory cytokines is defined as cytokine storm, which leading to multiple organs dysfunction. Our current study also explained the close link between cytokine level and organ failure. Therefore, according to the special immune profiles occurred in severe and critical COVID-19 patients, enhancing lymphocytes and inhibiting inflammation are the promising strategies for treatment of these COVID-19 patients.

Nevertheless, B cells exhibited a relatively opposite phenomenon from other lymphocytes, revealing that B cells are little impaired, even activated and amplified in partial patients, which could be the reactive activation of virus infection, or be the occurrence of B cell super antigen induced by novel coronavirus promotes immune resistance and suppression.

## Supplementary information

Supplementary materials
