# Supplementary material for: Immune characteristics of severe and critical COVID-19 patients
Source: Signal Transduct Target Ther. 2020 Aug 31;5:179. doi: 10.1038/s41392-020-00296-3 (PMC7456639; doi:10.1038/s41392-020-00296-3)
Supplement: Supplementary file 1 — Supplementary materials [file 41392_2020_296_MOESM1_ESM.docx]

Supplementary Materials for

Immune characteristics of severe and critical COVID-19 patients

Li Yang, Jianjun Gou, Jianbo Gao, Lan Huang, Zhiqiang Zhu, Shaofei Ji, Hongchun Liu, Lihua Xing*, Mengying Yao*, Yi Zhang*

Correspondence to: [yizhang@zzu.edu.cn](mailto:yizhang@zzu.edu.cn), [xinglihua95088@163.com](mailto:xinglihua95088@163.com), zdyfyricu@hotmail.com

**This file includes:**

Figures. S1 to S3

Table S1

Captions for Figures. S1 to S3


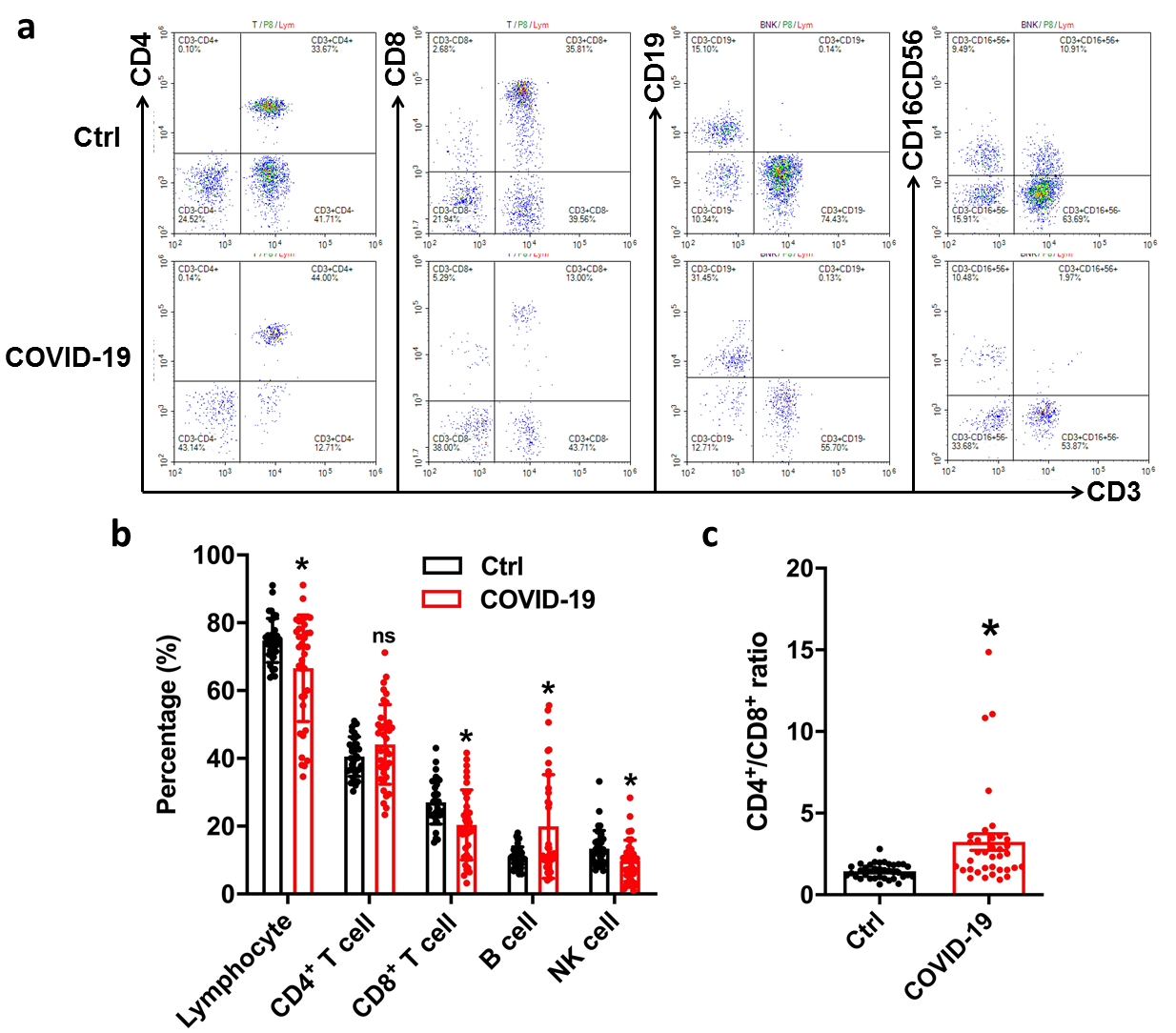


Figure S1. Comparison of lymphocyte subpopulations between COVID-19 patients and healthy donors. a. Lymphocyte subpopulations in the peripheral blood of COVID-19 (n=36) and control (healthy donor, n=36) groups were evaluated by flow cytometry. Representative analysis from one case is shown. b. The percentages of total lymphocytes, CD4^+^ T, CD8^+^ T, B and NK cells in COVID-19 and control groups were analyzed. c. CD4^+^/CD8^+^ T cell ratios in COVID-19 and control groups were analyzed. Data are represented as means ± SE. ns = non-significant, * = *P* < 0.05.


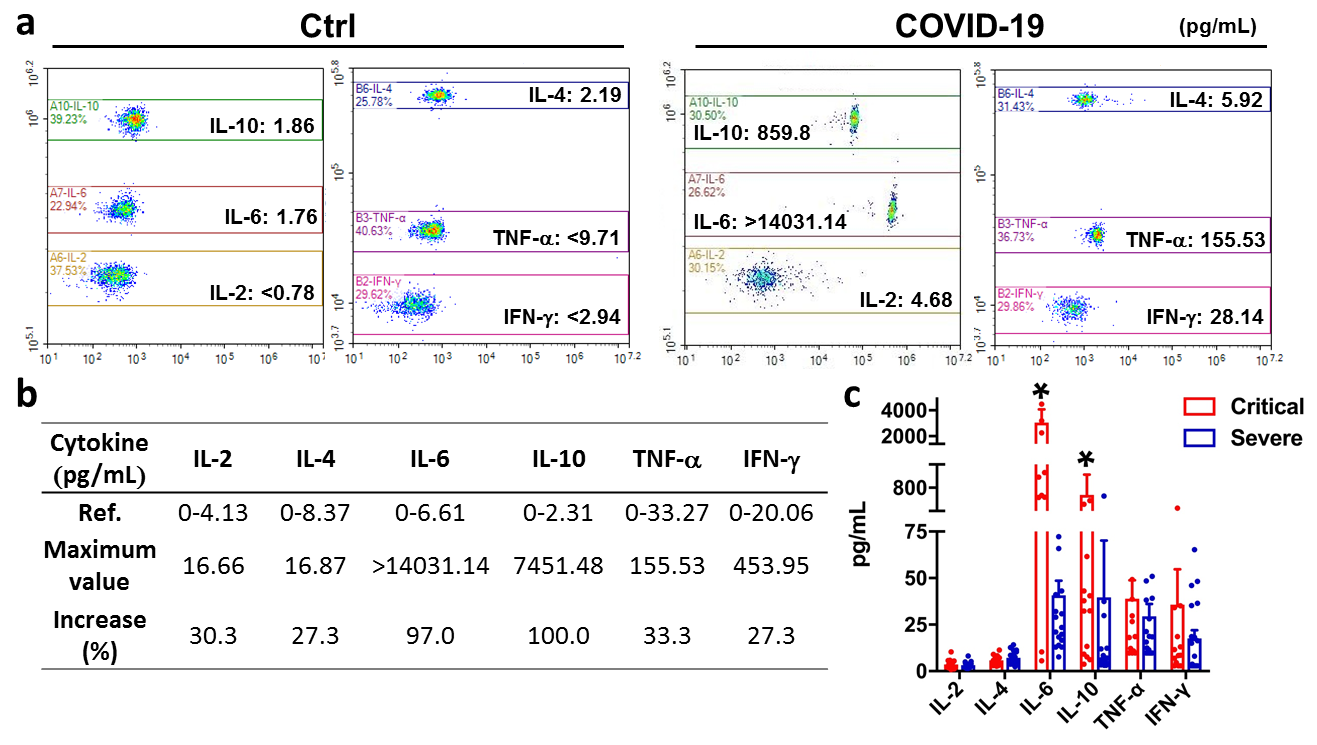


Figure. S2. Comparison of cytokines between COVID-19 patients and healthy donors. a. Levels of cytokines (IL-2, IL-4, IL-6, IL-10, TNF-α and IFN-γ) in the peripheral blood of COVID-19 (n=36) and control (healthy donor, n=36) groups were evaluated by flow cytometry. Representative analysis from one case is shown. b. The details of maximum value of cytokines, percentages of patients with increased cytokines were presented. c. Levels of cytokines (IL-2, IL-4, IL-6, IL-10, TNF-α and IFN-γ) in the peripheral blood of critical and severe COVID-19 patients were evaluated by flow cytometry, presented as a histogram. Data are represented as means ± SE. * = *P* < 0.05.


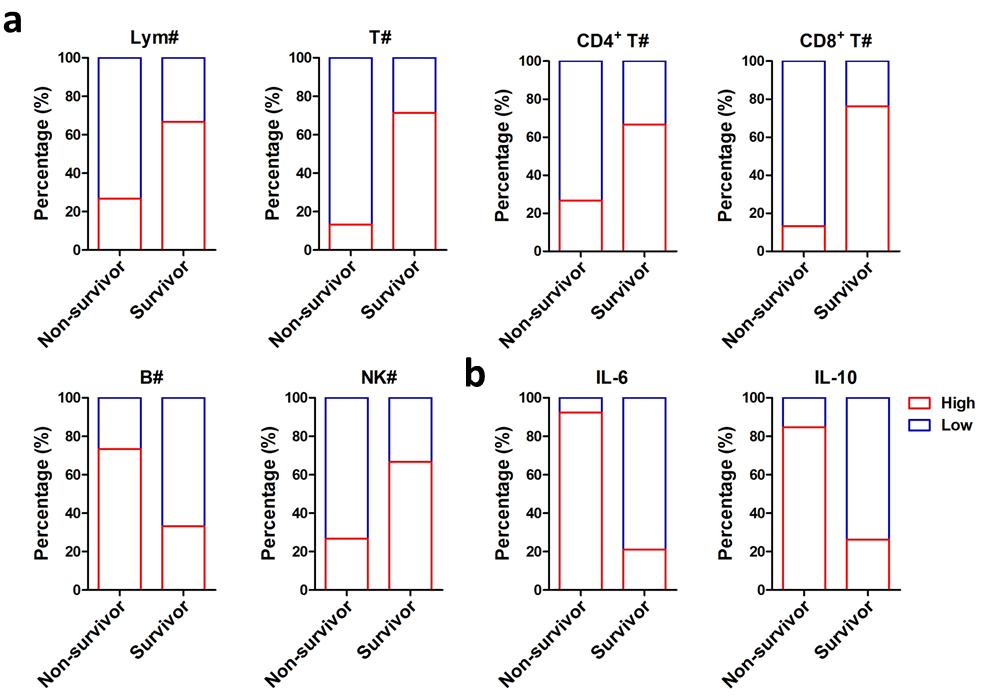


Figure. S3. Immune characteristics are closely correlated with disease progression of COVID-19. The levels of lymphocytes or cytokines from CVOID-19 patients were analyzed at the median day 28 after disease onset. a. The percentages of non-survivors or survivors with high and low levels of lymphocyte subpopulations. b. The percentages of non-survivors or survivors with high and low levels of IL-6 or IL-10.

Table S1. Details of microbiota infected in these COVID-19 patients.

| **No.** | **Microbiota name** | **Patient**  **number (n)** |
| --- | --- | --- |
| 1 | Baumanii | 7 |
| 2 | Klebsiella pneumoniae | 4 |
| 3 | Enterobacter cloacae complex | 2 |
| 4 | Stenotrophomonas maltophilia | 2 |
| 5 | Candida famata | 1 |
| 6 | Candida glabrata | 1 |
| 7 | Candida albicans | 1 |
| 8 | Enterococcus | 1 |
| 9 | Burkholderia cepacia | 1 |
| 10 | Pseudomonas aeruginosa | 1 |
| 11 | Saccharomyces cerevisiae | 1 |
